# Recent Advances in Photonic Crystal Fiber-Based SPR Biosensors: Design Strategies, Plasmonic Materials, and Applications

**DOI:** 10.3390/mi16070747

**Published:** 2025-06-25

**Authors:** Ayushman Ramola, Amit Kumar Shakya, Vinay Kumar, Arik Bergman

**Affiliations:** 1 Department of Electrical and Electronics Engineering, Ariel University, Ariel 40700, Israel; ayushmanr@ariel.ac.il (A.R.); arikb@ariel.ac.il (A.B.); 2 Department of Electronics and Communication Engineering, G.L. Bajaj Institute of Technology & Management, Knowledge Park III, Greater Noida 201306, India; vinay.kumar@glbitm.ac.in

**Keywords:** photonic crystal fiber, refractive index, surface plasmon resonance, plasmonic materials

## Abstract

This article presents a comprehensive overview of recent advancements in photonic crystal fiber (PCF)-based sensors, with a particular focus on the surface plasmon resonance (SPR) phenomenon for biosensing. With their ability to modify core and cladding structures, PCFs offer exceptional control over light guidance, dispersion management, and light confinement, making them highly suitable for applications in refractive index (RI) sensing, biomedical imaging, and nonlinear optical phenomena such as fiber tapering and supercontinuum generation. SPR is a highly sensitive optical phenomenon, which is widely integrated with PCFs to enhance detection performance through strong plasmonic interactions at metal–dielectric interfaces. The combination of PCF and SPR technologies has led to the development of innovative sensor geometries, including D-shaped fibers, slotted-air-hole structures, and internal external metal coatings, each optimized for specific sensing goals. These PCF-SPR-based sensors have shown promising results in detecting biomolecular targets such as excess cholesterol, glucose, cancer cells, DNA, and proteins. Furthermore, this review provides an in-depth analysis of key design parameters, plasmonic materials, and sensor models used in PCF-SPR configurations, highlighting their comparative performance metrics and application prospects in medical diagnostics, environmental monitoring, and chemical analysis. Thus, an exhaustive analysis of various sensing parameters, plasmonic materials, and sensor models used in PCF-SPR sensors is presented and explored in this article.

## 1. Introduction

Over the years, various types of optical fibers have been designed, developed, and fabricated to enhance fiber performance while reducing fabrication costs [1]. These fibers can be used for several applications, from sensing to communication [2]. Among these, photonic crystal fiber (PCF) has emerged as a specialized subset in a groundbreaking technology, offering significant advantages over conventional optical fibers [3]. PCF has attracted widespread interest from researchers due to its highly customizable design and versatile applications. Unlike traditional optical fibers, PCF overcomes structural limitations through its unique microstructured geometrical air-hole arrangement. Light propagation in the PCF occurs through two primary mechanisms. The first mechanism is index guiding, where the core has a higher refractive index (RI) than the cladding, and the second one is the photonic bandgap (PBG) effect, where light is confined despite the core having a lower RI than the surrounding cladding [4,5].

Key performance parameters of PCF include birefringence, nonlinearity, effective mode area, and confinement loss, all of which can be optimized by modifying the fiber’s geometric parameters, such as air-hole diameter (d), pitch (Λ), and thickness of plasmonic materials [6,7]. To achieve specific optical properties, such as zero dispersion, minimal propagation loss, or enhanced nonlinearity, PCF structures can be engineered into various air-hole lattice geometries, which include circular lattice [8], elliptical lattice [9], square lattice [10], and hexagonal arrangements [11]. Introducing high-nonlinearity liquid into PCF enhances soliton generation due to thermal effects and facilitates supercontinuum generation by increasing mode confinement [12]. Additionally, dispersion characteristics can also be precisely controlled by employing elliptical air holes, which enhances birefringence behavior in the sensor by carefully adjusting the microstructure’s shape and size to achieve flattened dispersion.

In recent years, numerous sensor designs based on one-dimensional photonic crystals (1D-PCs) have been fabricated. These structures consist of alternating layers of high- and low-dielectric materials arranged in a periodic pattern. Photonic crystals have the liberty to be fabricated in one-dimensional (1D) [13], two-dimensional (2D) [14], and three-dimensional (3D) [15] configurations, each offering unique optical properties. Their performance can be further enhanced by incorporating 2D materials like graphene, which improves light–matter interactions (LMI). Photonic crystals feature PBGs that prevent electromagnetic waves from propagating in specific directions. This unique ability to control light propagation has made photonic crystals highly relevant in various fields, including waveguiding, polarization conversion, biosensing, and optical filtering applications [16,17,18,19].

The integration of surface plasmon resonance (SPR) with PCF has opened new research avenues across multiple domains, including biosensing, bioimaging, environmental monitoring, and chemical sensing. PCF-based sensors leverage plasmon polaritons to facilitate the detection of unknown analytes. These plasmon polaritons are essentially surface waves that propagate along the dielectric–metal interface. Some prominent PCF-SPR sensor design models include the solid-core PCF-SPR sensor [20], hollow-core PCF-SPR sensor [21], internal metal deposition (IMD)-based sensor [22], external metal deposition (EMD)-based sensor [23], D-shaped sensor [24], asymmetric-air-hole-arranged sensor [25], slotted-shaped sensor [26], and hybrid-model-based sensor [27], which has a combination of various 2D materials.

To generate surface plasmons, several plasmonic materials, such as gold (Au), silver (Ag), aluminum (Al), copper (Cu), platinum (Pt), and palladium (Pd), play a prominent role [28,29,30]. These plasmons exist as free-charge oscillations at the metal–dielectric boundary, creating a strong localized electromagnetic field. Due to this field enhancement, plasmonic waves propagate along the interface and exhibit extreme sensitivity to changes in the surrounding environment. Resonance occurs when the wave vectors of the plasmonic and incident electromagnetic waves are phase-matched, leading to maximum energy transfer from the core mode to the plasmonic mode, which results in the generation of the SPR phenomenon [31]. These plasmonic materials possess different properties; for example, Au has high chemical stability and strong plasmonic response, Ag possesses a sharp resonance peak and higher sensitivity, Al is considered to have a strong plasmonic response in the UV region, Cu is a cost-effective alternative to Ag and Au, and Pt has high biocompatibility and stability [32,33]. Thus, plasmonic materials play a strong role in PCF-SPR sensor models.

PCF-SPR sensors are widely used for several applications across biomedical [34], environmental [35], and industrial domains [36]. In biomedical sensing, PCF-SPR sensors are extensively used for cancer detection [37], glucose monitoring [38], hemoglobin analysis [39], protein interaction studies [40], and DNA sensing [41], offering rapid, label-free, and highly selective detection of biomolecules. Their ability to detect minute RI changes makes them ideal for early-stage disease diagnosis, drug discovery, and environmental monitoring. These sensors are instrumental in detecting heavy metal ions like Pb^2+^, Cd^2+^, Hg^2+^, Cu^2+^, pesticides, and waterborne contaminants, ensuring real-time water quality assessment and pollution control [42,43]. Furthermore, PCF-SPR sensors are widely utilized in chemical sensing, where they facilitate the detection of toxic gases [44], volatile organic compounds (VOCs) [45], and industrial pollutants [46], aiding in air quality monitoring and hazardous gas detection [47]. In [48], a recent advancement in biosensors for dopamine detection, a key neurotransmitter with significant neurological and physiological roles, is presented. Traditional detection methods are costly and complex, prompting a shift toward miniaturized biosensors that offer high sensitivity, specificity, and cost-effectiveness. The integration of biomaterials and nanomaterials has significantly improved detection capabilities, with new biosensor generations utilizing aptamer receptors and organic electrochemical transistors (OECTs) achieving detection limits in the pico- to femto-molar range, aligning with clinical needs (0.1–10 nM). It also highlights an emerging technology that incorporates natural dopamine receptors, such as G protein-coupled receptors, into biosensor designs, offering highly selective, single-molecule-level detection despite current technological complexities. In the food industry, PCF-SPR sensors play a crucial role in food safety assessment, identifying contaminants such as bacteria, allergens, and spoilage markers. They show promising applications in medical diagnostics and point-of-care testing, enabling compact, real-time sensing for personalized healthcare.

The novelty of this review article lies in its comprehensive integration of PCF-based SPR biosensing mechanisms with advanced material innovations and emerging analytical applications, a convergence that has not been adequately addressed in previous biosensing reviews. While numerous articles have discussed either PCF structures or SPR biosensors independently, this review uniquely emphasizes how tailored PCF geometries, such as hexagonal, kagome, or multi-core arrangements, enhance the sensitivity, specificity, and miniaturization of SPR-based biosensors, especially for biomedical, chemical, and environmental diagnostics. Moreover, this article systematically categorizes plasmonic material (e.g., Au, Ag, graphene, transparent conducting oxides (TCOs), transition metal dichalcogenides (TMDs), and other novel 2D materials) and their impact on mode coupling and resonance quality in PCF-SPR systems [49,50,51]. One of the key contributions of this review is the detailed discussion of the advantages and limitations of various plasmonic materials used in sensor models, enabling researchers to select appropriate materials based on specific application requirements. In contrast to conventional reviews, which mainly discuss bulk plasmonic sensors or fiber optics in isolation, this work presents a focused, design-to-application roadmap for PCF-SPR biosensors, thereby serving as a technical and strategic reference for researchers and engineers developing next-generation biosensing platforms.

The article is organized as Section 2 of the article, presents details description of the various sensing parameters used in the SPR sensors, Section 3 presents details about various plasmonic materials, Section 4 presents details regarding various geometrical models of PCF-SPR sensors, Section 5 presents current and future prospects in PCF-SPR sensors, finally Section 6 presents concluding remark on the performed study.

## 2. Classification of Sensing Parameters for SPR Sensors

Sensing parameters associated with the PCF-SPR sensors provide information about how well these devices can identify a particular analyte. Therefore, by optimizing structural modifications and material selection, these sensors can achieve enhanced selectivity and efficiency. These sensing parameters are expressed as follows.

- **Confinement loss (CL) (dB/cm):** CL refers to the attenuation of light due to leakage from the fiber core into the surrounding medium, primarily caused by the interaction of guided light with surface plasmons at the metal–dielectric interface. This loss is a critical factor affecting sensor performance, as it directly influences sensitivity and detection accuracy. It is mathematically expressed by Equation (1) [52,53].
  (1) $$Confinement\;Loss\;\left(CL\right)\left(\frac{dB}{cm}\right)=8.686\times k_{0}\times Im(n_{eff})\times 10^{4}$$
  where $k_{0}=\frac{2\pi }{\lambda }$ is a wave number and $Im(n_{eff})$ is the imaginary part of the effective mode index.
- **Birefringence (*B*):** Is a measure of the difference in the effective RI for two orthogonal polarization modes. Birefringence arises due to the asymmetry in the fiber structure, e.g., elliptical air holes, asymmetric core design, D-shaped fibers, or external coatings. The interaction between the guided light and surface plasmon waves (SPWs) also introduces birefringence. It is expressed by Equation (2) [54,55].
  (2) $$Birefringence\;\left(B\right)=\left|Re\left(n_{eff}^{x}\right)-Re(n_{eff}^{y})\right|$$
  where $Re\left(n_{eff}^{x}\right)$ and $Re(n_{eff}^{y})$ is the real part of the effective mode index for two different polarizations, i.e., TM pol. and TE pol., respectively.
- **Wavelength Sensitivity (WS):** Quantifies the shift in resonance wavelength (RW) per unit change in the external RI. It is expressed by Equation (3) [56,57].
  (3) $$Wavelenght\;Sensitivity\;(WS)=∆\lambda_{peak}/∆n_{a}\;\left[nm/RIU\right]$$
  where $∆\lambda_{peak}$ represents the wavelength variation between the peak, and $∆n_{a}$ is the difference between two successive analytes. WS is calculated using the wavelength interrogation technique.
- **Amplitude Sensitivity (AS):** AS refers to how the amplitude of the sensor’s output signal changes in response to variations in the RI of the medium surrounding the fiber. This can be particularly useful in detecting small shifts in the environment, such as changes in the concentration of chemical or biological species. It can be expressed by Equation (4) [58,59].
  (4) $$Amplitude\;Sensitivity\;\left(AS\right)=-\frac{1}{\alpha \left(\lambda ,\;n_{a}\right)}\frac{\partial \alpha \left(\lambda ,n_{a}\right)}{\partial n_{a}}[{RIU}^{-1}]$$
  where $\alpha \left(\lambda ,\;n_{a}\right)$ represents total CL, $\partial \alpha \left(\lambda ,n_{a}\right)$ represents the change in CL for different wavelengths, and $\partial n_{a}$ represents the change in RI.
- **Sensor Resolution (SR):** SR refers to the smallest change in the RI or RW that the sensor can detect reliably. It can be expressed by Equation (5) [60,61].
  (5) $$Sensor\;resolution\;(SR)={∆n}_{a}\frac{∆\lambda_{min}}{∆\lambda_{peak}}[RIU]$$
  where $∆\lambda_{min}$ represents minimum spectral resolution and is considered 0.1 nm for resolution calculation.
- **Figure of Merit (FOM):** It is a critical performance metric that quantifies the sensor’s sensitivity and resolution in detecting changes in the RI of the surrounding medium. It combines both the sensitivity to RI changes and the sharpness of the resonance peak, providing a comprehensive measure of the sensor’s overall performance. It is expressed by Equation (6) [62,63].
  (6) $$Figure\;of\;Merit\;(FOM)=\frac{ws}{FWHM}[{RIU}^{-1}]$$
  where FWHM represents the full wave’s half maximum.
  Similarly, there are PRISM-based SPR sensors. These sensors’ performance also depends upon parameters like sensitivity (S), FOM, and detection accuracy/signal-to-noise ratio (DA/SNR). Details regarding these parameters are expressed as follows.
- **Sensitivity (S):** In the PRISM-based configuration, sensor sensitivity (*S*) refers to the ability of the sensor to detect small changes in the RI of the surrounding medium based on the shift in the resonance angle (Δ*θ*) or wavelength (Δλ). Specifically, the sensitivity quantifies how much the resonance condition changes when the angle or wavelength at which SPR occurs changes, with the response to a change in the RI of the surrounding medium ($∆n_{s}$). It is expressed by Equation (7) [64,65].
  (7) $$S=\frac{∆\theta_{res}}{∆n_{s}}\left({}^{\circ}/RIU\right)$$
- **Figure of Merit (FOM):** In the context of the PRISM-based SPR, sensor FOM is a crucial metric that combines the sensitivity of the sensor with the sharpness of the resonance peak. The FOM quantifies the sensor’s overall performance in terms of its ability to detect small changes in the RI of the surrounding medium, while accounting for the quality of the SPR signal. It is expressed by Equation (8) [66,67].
  (8) $$FOM=S\times DA(\frac{1}{RIU})$$
- **Angular detection sensitivity (DA)**: This parameter is a way to quantify the angular sensitivity of the SPR sensor based on the sharpness of the resonance curve. It indicates how much angular shift (in degrees) a sensor can detect per unit change in the RI of the surrounding medium. It is expressed by Equation (9) [68,69].
  (9) $$DA=\frac{1}{FWHM}(1/{}^{\circ})$$
  Similarly, various other sensor performance parameters can be evaluated by studying the SPR phenomenon in the PCF-SPR sensor and PRISM-based SPR sensors.

## 3. Background of Plasmonic Materials Used in PCF-SPR Sensors

Plasmonic materials play a crucial role in the functioning of PCF-SPR sensors, as they enable the excitation of SPWs, which are responsible for the sensor’s sensitivity to RI changes in the surrounding medium [70]. The interaction between incident light and the free electrons of the plasmonic material forms the basis of SPR sensing. SPR is an optical phenomenon where the incident light excites collective oscillations of free electrons at the interface of a metal and a dielectric surface. This results in the generation of an evanescent field that is highly sensitive to the RI changes in the analyte [71]. The guided light in the PCF will work in the following steps.

- Light is guided through the air holes of the PCF.
- The plasmonic material is coated on the inner walls of the fiber holes or externally on a flat-polished fiber surface.
- The evanescent field from the core mode penetrates the plasmonic layer, exciting surface plasmons at a specific RW.
- A change in the analyte’s RI shifts the RW, allowing precise sensing.

Besides plasmonic materials, the Sellmeier equation is also used to model the SPR phenomenon. It is widely used in PCF-SPR sensors to model the wavelength-dependent RI of dielectric materials, including the fiber core, analyte, and plasmonic coatings. The Sellmeier equation plays a crucial role in PCF-SPR sensors by providing an accurate mathematical model for the wavelength-dependent RI of fiber materials, plasmonic coatings, and analytes. It ensures precise phase-matching, enhances SPR sensitivity, and optimizes sensor performance. The Sellmeier equation is expressed by Equation (10) [72,73].

(10) $$n^{2}\left(\lambda \right)=1+\sum_{i}\frac{B_{i}\lambda^{2}}{\lambda^{2}-C_{i}}$$

where λ is the operating wavelength, $B_{i}$ and $C_{i}$ are the experimentally determined values known as the Sellmeier coefficients. The values of the Sellmeier coefficients vary for different materials, like the Sellmeier coefficients for commonly used fused silica are expressed as $B_{1}=0.696166300$, $B_{2}=0.407942600$, $B_{3}=0.897479400$, $C_{1}=4.67914826\times 10^{-3}\;{\mu m}^{2}$, $C_{2}=1.35120631\times 10^{-2}\;{\mu m}^{2}$, and $C_{3}=97.9340025\;{\mu m}^{2}$ [74]. Similarly, for the magnesium fluoride (MgF_2_), which is considered a good alternative to Au, the values of the Sellmeier coefficients are expressed as $B_{1}=0.48755108$, $B_{2}=0.39875031$, $B_{3}=2.3120353$, $C_{1}=0.001882178\times 10^{-3\;}{\mu m}^{2}$, $C_{2}=0.008951888\times 10^{-2}{\mu m}^{2}$, and $C_{3}=566.13559\;{\mu m}^{2}$ [75]. Likewise, the values of the Sellmeier coefficients vary for different plasmonic materials.

### 3.1. Optical Properties of Plasmonic Material Gold (Au)

Au is considered one of the most widely used plasmonic materials due to its excellent plasmonic properties and chemical stability. Au is known for its strong plasmonic resonance, especially in the visible to near-infrared (VNIR) range [76]. SPPs are excited when the incident light interacts with the free electrons at the metal–dielectric interface, leading to resonance phenomena. The RW of Au typically falls around 520–600 nm, depending on the surrounding medium and geometrical configuration [77]. Au supports SPPs that propagate along the surface of the metal when excited by incident light. The SPPs are highly sensitive to changes in the RI of the surrounding medium, which is the principle behind SPR sensing. The dielectric constant of Au is expressed by the Drude–Lorentz model represented by Equation (11) [78].

(11) $$\varepsilon_{Au}=\varepsilon_{\infty }-\frac{\omega_{D}^{2}}{\omega \left(\omega -j\gamma_{D}\right)}-\frac{∆\varepsilon {Ω}_{L}^{2}}{\left(\omega^{2}-{Ω}_{L}^{2}\right)-j{ɼ}_{L}\omega }$$

where $\varepsilon_{Au}$ is defined as the permittivity of Au, $\varepsilon_{\infty }\;$ is the high frequency dielectric constant, and ∆ε  is the weighing factor. The angular frequency of light is ω=2π/λ. Plasma frequency and damping frequency are defined as $\omega_{D}=4227.2\;THz\;$ and $\gamma_{D}=31.84\;\pi \;THz$. Finally, ${Ω}_{L}=1300.14\;\pi \;THz$ and ${ɼ}_{L}=209.72\;\pi \;THz$.

*Advantages*

- Au exhibits strong SPR in the VNIR regions, making it ideal for biosensing and optical applications.
- It is highly resistant to oxidation and corrosion, unlike Ag or Cu, ensuring long-term stability in plasmonic devices.
- It is non-toxic and widely used in biomedical applications such as biosensors, drug delivery, and imaging.

*Disadvantages*

- Au has higher intrinsic losses in the VNIR range compared to Ag, leading to reduced efficiency in some plasmonic applications.
- It is costly compared to other plasmonic materials like Ag and Al, increasing the overall cost of plasmonic devices.
- It performs well in the VNIR spectrum but is less effective in the ultraviolet (UV) region due to high absorption.

### 3.2. Optical Properties of Plasmonic Material Silver (Ag)

Ag is one of the most widely used plasmonic materials due to its exceptional optical properties. It exhibits strong surface SPR in the VNIR regions. The dielectric constant of Ag is expressed by the Drude–Lorentz model and presented by Equation (12) [78,79].

(12) $$\;\varepsilon_{r}\left(\omega \right)=1-\frac{{Ω}_{p}^{2}}{\omega (\omega -i{ɼ}_{0})}+\sum_{j=1}^{k}\frac{f_{j}\omega_{p}^{2}}{\left(\omega_{j}^{2}-\omega^{2}\right)+i\omega {ɼ}_{j}}$$

where $\omega_{p}$ = 9.01 represents plasma frequency, $\omega_{j}$ represents the interband transition frequency, $f_{j}\;$ represents the oscillator strength, lifetime $1/{ɼ}_{j}$, ${Ω}_{p}$ is the plasma frequency, and the damping coefficient is ${ɼ}_{0}.$

*Advantages*

- Ag is considered to have one of the highest plasmonic performances among noble metals due to its low optical losses and strong SPR properties in the VNIR spectrum.
- Compared to Au, Ag exhibits lower absorption losses, resulting in higher field enhancement and better performance in plasmonic devices.
- It supports plasmonic resonance from the UV to the NIR region, making it versatile for different optical applications.

*Disadvantages*

- Ag readily oxidizes in air or aqueous environments, forming silver sulfide (Ag_2_S), which degrades its plasmonic properties over time.
- Although Ag is used in several biomedical applications, its potential cytotoxicity limits its use in some biological systems.
- Its plasmonic response can shift due to thermal effects, making it less suitable for high-temperature applications.

### 3.3. Optical Properties of Plasmonic Material Aluminum (Al)

Al is a strong plasmonic material with unique optical and electronic properties, making it highly relevant in UV, visible, and NIR SPR applications, unlike plasmonic materials like Au and Ag. Al offers a higher plasma frequency, enabling plasmonic effects in the UV region [80]. The dielectric constant of Al is expressed by the Drude–Lorentz model, as expressed by Equation (13) [81].

(13) $$\varepsilon \left(\omega \right)=\varepsilon_{\infty }-\frac{\omega_{p}^{2}}{\omega^{2}+i\gamma_{\omega }}$$

where $\varepsilon_{\infty }=1$, $\omega_{p}=1.75\times 10^{16}\;rad/s$ is the plasma frequency, $\gamma =1.22\times 10^{14}\;rad/s$ is the damping coefficient.

*Advantages*

- Al supports surface plasmons in the deep UV region (200–400 nm), making it suitable for biochemical sensing, DNA analysis, and virus detection.
- It is much cheaper than Au and Ag, making it attractive for low-cost SPR sensors.

*Disadvantages*

- Al is a strong plasmonic material in the UV, but it has high losses in the visible range.
- It forms a thick oxide layer, altering its plasmonic properties and resulting in oxidation loss.

Besides these popular plasmonic materials, some other materials like Cu [82], Pd [83], Pt [84], graphene [85], titanium dioxide (TiO_2_) [86], and tantalum pentoxide (Ta_2_O_5_) [87] are widely used in the PCF-SPR sensor models. The RI of these materials is expressed as follows.

The RI of TiO_2_ is expressed by Equation (14) [86].

(14) $$n_{TiO_{2}}=\sqrt{5.913+\frac{0.2441}{\lambda^{2}-0.0803}}$$

The RI of Ta_2_O_5_ is expressed by Equation (15) [87].

(15) $$n_{{Ta}_{2}O_{5}}=1.88+\frac{178.4\times 10^{2}}{\lambda^{2}}+\frac{52.7\times 10^{7}}{\lambda^{4}}$$

The RI of graphene is expressed by Equation (16) [85].

(16) ng=3+i5.446×λ3

where *λ* is the operating wavelength of the designed PCF-SPR sensor.

### 3.4. Optical Properties of Transparent Conductive Oxides (TCOs)

TCOs are a class of low-loss plasmonic materials that combine optical transparency in the visible spectrum with high electrical conductivity. Unlike traditional plasmonic metals like Ag, Au, Al, etc., TCOs offer tunable optical properties, making them attractive alternatives for PCF-SPR sensor applications. These materials are particularly useful for SPR sensing in NIR and offer several advantages over traditional metals. Some prominent TCO materials that exhibit plasmonic behavior in the NIR and mid-infrared (MIR) regimes [88] are listed as indium tin oxide (ITO) [89], aluminum-doped zinc oxide (AZO) [90], gallium-doped zinc oxide (GZO) [91], fluorine-doped tin oxide (FTO) [92], cadmium oxide (CdO) [93], etc. The RI of TCOs, such as ITO, AZO, GZO, FTO, and CdO, follows the Drude–Lorentz model for plasmonic materials. The general expression of the Drude–Lorentz model for TCOs is expressed by Equation (17) [94].

(17) $$\varepsilon \left(\omega \right)=\varepsilon_{\infty }-\frac{\omega_{p}^{2}}{\omega^{2}+i\gamma \omega }+\sum_{j}\frac{S_{j}\omega_{j}^{2}}{\omega_{j}^{2}-\omega^{2}-i\Gamma_{j}\omega }$$

where $S_{j}$, $\omega_{j}$ and $\Gamma_{j}$ represent the Lorentz oscillator strength, resonant frequency, and damping term for interband transitions. Some of the important features of TCOs, which are why they are preferred over conventional plasmonic materials, are expressed as follows.

- TCOs allow tunable optical customization of PCF-SPR resonance by adjusting carrier concentration.
- Compared to plasmonic materials like Au and Ag, TCOs exhibit reduced propagation losses.
- They have a broadband plasmonic response and are effective across UV, visible, NIR, and IR ranges.
- They are extensively used for detecting minute RI changes, enhancing biosensing, and chemical sensing.
- They offer economic and stable alternatives to traditional plasmonic metals.

Nowadays, researchers have presented PCF-SPR sensor models based on MnO_2_. Yan et al. [95] presented a dual-channel SPR fiber sensor for the rapid and sensitive detection of glutathione (GSH) and RI changes. The sensor features a novel combination of Au–MnO_2_ thin film in Channel 2 for GSH detection, leveraging the dissolution of MnO_2_ upon interaction with GSH. Channel 1 uses an ITO-Ag thin film for RI sensing and can also detect high-concentration GSH. The sensor achieves a GSH sensitivity of –2.361 nm/mM (0.005–50 mM range) and an RI sensitivity of 1704.252 nm/RIU (1.331–1.3895 RIU range). In high GSH concentrations (50–600 mM), a sensitivity of 0.095 nm/mM is observed. The MnO_2_ film boosts detection sensitivity by 25.663 times. The two channels operate independently, offering high selectivity, enhanced sensitivity, and strong potential for biological and environmental applications.

### 3.5. Optical Properties of Emerging Materials MXenes

MXenes are 2D transition metal carbides, nitrides, and carbonitrides. The general expression to represent MXenes is M_n+1_X_n_T_x_, where M = Transition metal (Ti, Nb, V, Mo, etc.), X = carbon and/or nitrogen, and T_x_ = surface termination groups (-OH, -O, -F) [96,97]. These materials exhibit strong plasmonic behavior due to metallic conductivity, high charge carrier density, and unique optical properties, making them promising alternatives to traditional metals in PCF-SPR sensors [98]. MXenes support localized surface plasmon resonances (LSPR) in the visible to MIR range, which leads to strong light confinement and enhanced plasmonic sensitivity, beneficial for RI sensing applications [99].

*Advantages*

- MXenes are a relatively stable material in various environments.
- Plasmonic resonances extend from visible to MIR, making them ideal for biosensing, chemical detection, and environmental monitoring.
- Their plasmonic response can be modified via surface chemistry, offering better control over resonance conditions in PCF-SPR sensors.
- They can be combined with Au, Ag, or graphene for hybrid plasmonic structures with enhanced sensitivity and stability.

*Disadvantages*

- MXenes tend to oxidize in air and aqueous environments, leading to a loss of plasmonic performance.
- Their synthesis involves etching MAX phases, which require controlled chemical processing.
- Maintaining layer thickness uniformity is crucial for consistent plasmonic properties.

### 3.6. Optical Properties of Emerging Materials: Transition Metal Dichalcogenides (TMDs)

TMDs are a class of 2D materials composed of a transition metal (Mo, W) sandwiched between chalcogen atoms (S, Se, Te) [100]. Some of the prominent TMDs plasmonic materials are molybdenum disulfide (MoS_2_) [101], tungsten disulfide (WS_2_) [102], molybdenum diselenide (MoSe_2_) [103], and tungsten diselenide (WSe_2_) [104]. TMDs like MoS_2_ and WS_2_ exhibit strong LMI, enabling LSPR that can be harnessed for highly sensitive detection. Their large excitonic effects and monolayer nature provide enhanced optical responses, which are crucial for improving sensor performance. TMDs offer tunable band gaps depending on the number of layers, from an indirect bandgap (bulk) to a direct bandgap (monolayer). This tunability allows control over the plasmonic resonance frequency, making them suitable for multi-wavelength sensing. By adjusting the layer thickness or applying strain, the optical properties of TMDs can be tailored to match the required sensing range in PCF-SPR sensors.

*Advantages*

- The high optical absorption and tunability of TMDs make them ideal for low-concentration sensing, especially for biomolecules, gases, and environmental pollutants.
- TMDs, especially MoS_2_ and WS_2_, show excellent chemical stability, making them more robust than traditional plasmonic materials like Au and Ag, which are susceptible to oxidation and corrosion.
- TMDs can be integrated into flexible substrates, offering potential for wearable PCF-SPR sensors that are lightweight and flexible for applications in biomedical and environmental monitoring.
- TMDs are particularly well-suited for biosensing due to their large surface area and biocompatibility, allowing for the detection of proteins, nucleic acids, and small molecules with high accuracy.

*Disadvantages*

- Producing high-quality monolayers of TMDs without defects is challenging. Imperfections can degrade the plasmonic properties and can affect sensor performance.
- Large-scale integration of TMDs into PCF-SPR sensors requires precise layer control and sophisticated fabrication techniques.
- The optical properties of TMDs are highly sensitive to strain, which can lead to performance variation in PCF-SPR sensors under environmental changes or mechanical stress.
- While TMDs are highly promising, their commercial availability for large-scale sensor applications is still in its early stages. The materials may not yet be as widely accessible as conventional plasmonic metal.

Besides, it is important to note that TMDs also present ferroelectric properties. Dushaq et al. [105] presented the strong electro-refractive tuning capabilities of multilayer CuCrP_2_S_6_ (CCPS), a ferroionic 2D material, integrated into silicon photonics microring resonators for near-infrared (NIR) applications. The device achieves a RI modulation of ~2.8 × 10^−3^ RIU with low optical loss and high modulation efficiency (0.25 V·cm), outperforming previous TMD-based phase shifters. It shows a consistent blue shift in resonance and a polarization-dependent response between TE and TM modes. These features make CCPS promising for compact, efficient, and tunable optical components in applications such as optical switching, phased arrays, environmental sensing, imaging, metrology, and neuromorphic systems.

## 4. Classification of PCF-SPR Sensor Based on Design Configurations

PCF-SPR sensors can be classified into various models based on their structural design. Each model geometry offers unique advantages in terms of sensing behavior. PCF-SPR sensors offer a wide range of structural and functional models, each optimized for specific applications. The choice of models depends on factors like sensitivity requirements, target analytes, and fabrication feasibility. With continuous advancements in nanomaterials, fiber optics, and hybrid models integrating 2D materials and novel plasmonic coatings are set to revolutionize SPR-based sensing technologies. Some of the prominent PCF-SPR sensor design includes sensor based on solid-core PCF [106], hollow-core PCF [107], elliptical core PCF [108], slotted-PCF [109], conventional cladding-coated PCF [110], multi-air-hole cladding PCF [111], D-shaped PCF [112], IMD-based PCF [113], and EMD-based PCF [114]. Other kinds of fiber include index-guiding PCF-SPR sensors and PBG PCF-SPR sensors.

### 4.1. Solid-Core PCF-SPR Sensor

**Figure 1:**
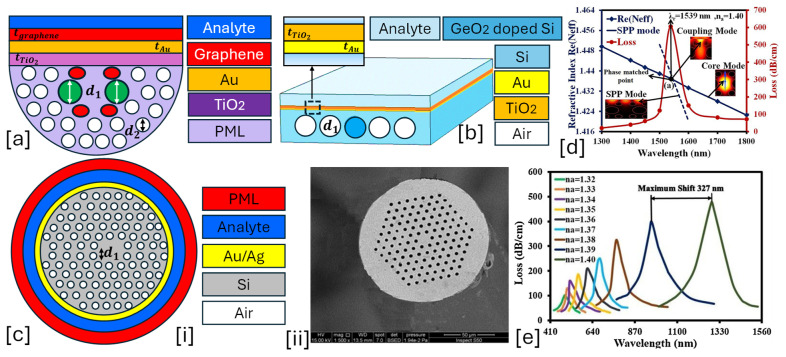
Solid core PCF-SPR sensors. (**a**) D-shaped Au-graphene-TiO_2_-coated PCF [117]. (**b**) Non-conventional GeO_2_ doped Au-TiO_2_-coated PCF [118]. (**c**) [i] EMD-based PCF with hexagonal lattice of air holes [119]; [ii] SEM image of the fabricated PCF [119]. (**d**) presents the optical dispersion relationship between the core mode and SPP mode for the sensor model presented in (**a**); these modes intersect each other at a particular wavelength, which is known as the phase-matching condition [117]. (**e**) represents the behavior of the analytes for RI varying from 1.32 to 1.49 RIU for the sensor model presented in (**a**) [117].

A solid-core PCF-SPR sensor leverages the unique properties of PCFs and SPR to detect biomolecules, chemicals, and RI variations in a highly sensitive manner. The solid-core PCF guides light via modified total internal reflection (m-TIR) or index-guiding mechanisms. The fiber core is surrounded by an air-hole cladding, which alters the modal properties. The SPR effect is exploited by monitoring the RW shift as the RI of the external medium changes. The resonance condition shifts when target analytes bind to the sensor surface, making it highly effective for biochemical sensing, gas sensing, and environmental monitoring [115,116]. Figure 1 presents 2D models of the various types of solid core PCF-SPR sensor models proposed by various researchers based on different structural geometries and plasmonic materials.

Some prominent features of these types of sensors are listed as follows.

- Achieves high sensitivity by optimizing plasmonic layer thickness and structure.
- The unique structure supports strong mode confinement and effective light–plasmon interaction.
- Allows tunable sensing by selecting different plasmonic materials and fiber geometries, e.g., dual-core, D-shaped, or selectively coated structures.
- Can be optimized for VNIR sensing applications.

### 4.2. Hollow-Core PCF-SPR Sensor

A PCF-SPR sensor is an advanced optical sensing platform that utilizes a hollow-core guiding mechanism in conjunction with SPR for high-sensitivity RI sensing. Unlike solid-core PCF-SPR sensors, where light propagates through a silica core, hollow-core PCFs guide light through an air-filled core, enhancing interaction with analytes and the plasmonic layer [120]. Hollow-core PCF-SPR sensors typically feature a large central air hole in place of a solid silica core. The surrounding microstructured cladding, consisting of periodic air holes, helps confine and guide light through the fiber via the PBG effect or anti-resonant guiding (ARG) mechanism [121,122].

**Figure 2:**
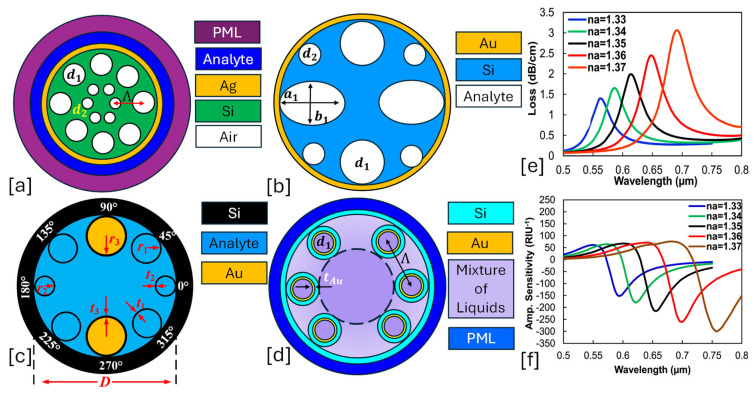
Hollow core PCF-SPR sensors. (**a**) Ag-coated dual lattice of hexagonal and octagonal air holes [123]. (**b**) Au-coated elliptical and circular air hole PCF [124]. (**c**) Octagonal lattice of three-dimensional air holes [125]. (**d**) Au-coated hexagonal lattice of air holes [126]. (**e**) represents the behavior of CL for analyte RI varying in the RI range of 1.33–1.37 RIU for the sensor model presented in (**a**) [123]. (**f**) represents the AS behavior for RI ranging from 1.33–1.37 RIU for the sensor model presented in (**a**) [123].

They allow direct interaction between the guided light and the analyte, enabling high sensitivity. A thin metal layer is coated inside the hollow core or on selected cladding holes to excite surface plasmon waves. A lattice of air holes ensures light confinement and minimizes propagation loss. Figure 2 presents 2D models of hollow core PCF-SPR sensor models having a combination of circular and elliptical air holes within the background materials.

Some prominent features of these types of sensors are listed as follows.

- Direct exposure of guided light to the analyte.
- Optimized microstructured cladding structure to ensure better light confinement.
- Multiple analytes can be detected simultaneously by functionalizing different sections of the core.
- Effective for low and high RI ranges and suitable for gas and liquid sensing.

### 4.3. Elliptical-Core PCF-SPR Sensor

**Figure 3:**
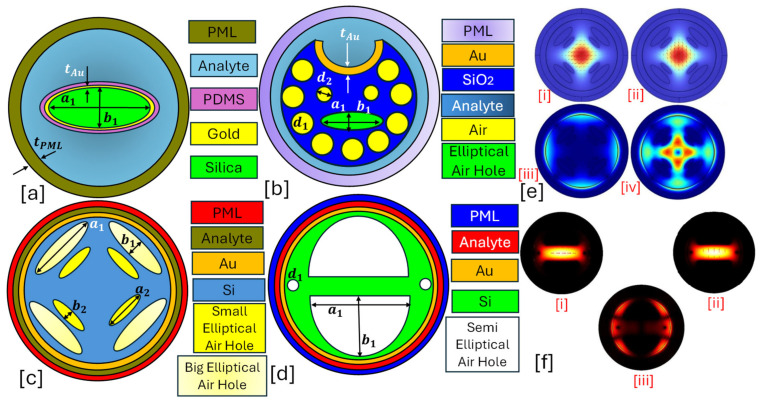
Elliptical-core PCF-SPR sensor. (**a**) Au-coated single elliptical air hole PCF [130]. (**b**) Au-coated slotted-PCF with elliptical and circular air holes [131]. (**c**) Au-coated PCF with dual-dimensional elliptical air hole [132]. (**d**) Au-coated dual semielliptical air hole PCF [133]. (**e**) [i] represents the x-polarized core mode for the sensor presented in (**c**) [132]; [ii] represents the y-polarized core mode for the sensor presented in (**c**) [132]; [iii] represents the SPP mode formation for the sensor presented in (**c**) [132]; [iv] represents the coupling mode formation for the sensor presented in (**c**) [132]. (**f**) Field distribution profile for the sensor model presented in (**d**) [133], [i] x-polarized core mode [133]; [ii] y-polarized core mode [133]; [iii] SPP mode [133].

An elliptical-core PCF-SPR sensor is a specialized optical sensing platform designed to enhance polarization-dependent sensing, mode confinement, and sensitivity. The elliptical core introduces birefringence, allowing precise control of light polarization and stronger interaction with the plasmonic layer, making it ideal for high-resolution RI sensing applications [127]. Elliptical-core sensors differ from conventional circular-core PCFs by having an asymmetrically shaped core, which influences the propagation characteristics of the fundamental and higher-order modes [128,129]. Figure 3 presents 2D models of elliptical core PCF-SPR sensors having circular, elliptical, and semielliptical air hole arrangements.

The main advantages of having an elliptical-core structure include the following.

- The introduction of birefringence enables enhanced light–plasmon coupling and reduces propagation loss.
- An array of air holes arranged in a hexagonal or square lattice is suitable for optimizing modal dispersion.
- A thin metal coating of plasmonic materials is deposited on selected cladding holes or the fiber surface to facilitate SPR excitation.
- The elliptical shape alters the evanescent field penetration, enhancing analyte interaction.
- The elliptical shape provides strong plasmonic interaction due to asymmetric evanescent field distribution.
- They enable selective excitation of SPR modes, reducing interference.
- Optimized core geometry minimizes loss while maintaining high confinement.

### 4.4. Slotted-Core PCF-SPR Sensor

**Figure 4:**
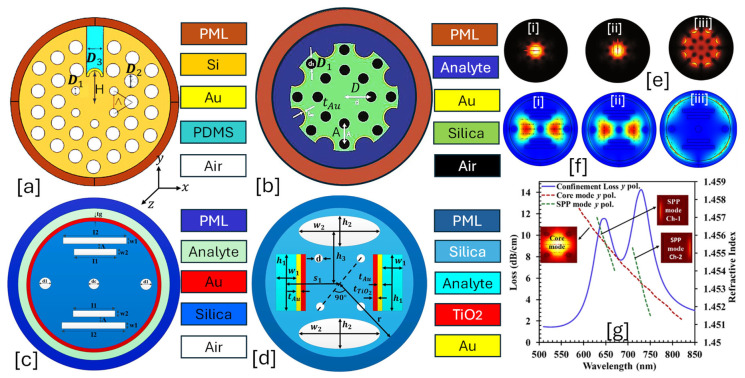
Slotted-ore PCF-SPR sensor. (**a**) Au-coated single slot PCF with a hexagonal lattice of two different dimensions of air holes [135]. (**b**) Au-coated PCF with octagonal slot in background material [136]. (**c**) Au-coated PCF with rectangular slot of two dimensions [137]. (**d**) Au-TiO_2_-coated PCF with elliptical shape slot in the background material [138]. (**e**) Mode profiles of the sensor presented in (**b**) [136], [i] x-pol. core mode [136]; [ii] y- pol. core mode [136], [iii] SPP mode [136]. (**f**) Mode profile of the sensor presented in (**c**) [137], [i] dual-core mode x-pol. [137]; [ii] dual-core mode y-pol. [137]; [iii] SPP mode [137]. (**g**) Optical field distribution profile for the sensor model presented in (**d**), having two peak CLs concerning Channel 1 and Channel 2, respectively [138].

A slotted-core PCF-SPR sensor is an advanced optical sensing platform designed to enhance light–analyte interaction by introducing a nano-slot in the fiber core. This configuration increases evanescent field exposure, resulting in higher sensitivity and improved sensing resolution [134]. The slotted-core PCF-SPR sensor features a unique nano-slot embedded within the core region, allowing direct exposure of the guided light to the analyte. Figure 4 presents 2D models of slotted-core PCF-SPR sensors having a variety of slots in the background materials. The main design components of this sensor model are expressed as follows.

- A narrow slot in the fiber core that enhances evanescent field interaction with the analyte.
- A hexagonal or rectangular lattice of air holes is suitable for light confinement.
- A thin metallic coating of plasmonic material is deposited inside the slot or on the surrounding cladding.
- The slot structure enables stronger SPR excitation, increasing resonance shifts.

Some prominent features of these sensor models are listed as follows.

- The nano-slot maximizes light–analyte interaction, enhancing detection accuracy.
- Optimized slot dimensions reduce leakage loss while maintaining strong plasmonic coupling.
- The slot can be engineered for specific analytes, making the sensor highly versatile.
- Functionalization of the slot allows for the simultaneous detection of multiple biomolecules.

### 4.5. Conventional Cladding-Coated PCF

**Figure 5:**
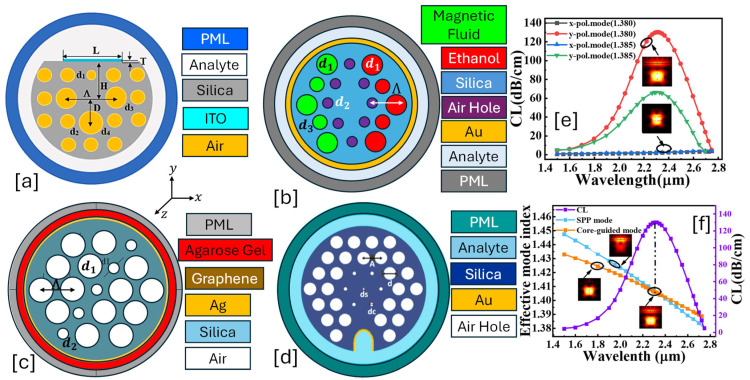
Conventional cladding-coated PCF. (**a**) Quasi-D-shaped ITO-coated PCF [140]. (**b**) EMD-based hexagonal shape PCF with three-dimensional air holes [141]. (**c**) Ag-coated PCF for Agarose gel detection based on two air hole geometries [142]. (**d**) Au-coated PCF having a hexagonal lattice of air holes and a semielliptical slot in the background material [143]. (**e**) x-polarization and y-polarization field distribution profile and CL behavior of the sensor presented in (**a**) [140]. (**f**) Optical field distribution of the SPP mode (blue) and core guided mode (orange) for the sensor presented in (**a**) [140].

A conventional cladding-coated PCF-SPR sensor is one of the most widely used PCF-SPR sensor configurations. It features a metallic-based plasmonic coating applied to the cladding region, allowing the guided light to interact with surface plasmons through evanescent field coupling [139]. This type of sensor is particularly useful for biosensing, chemical sensing, and environmental monitoring due to its simple fabrication and high sensitivity. Figure 5 presents 2D models of conventional cladding-coated PCF-SPR sensors. The cladding-coated PCF-SPR sensor consists of the following features.

- Depending on the design, the core can be solid (high index) or hollow (air-filled) to optimize mode confinement.
- A periodic arrangement of air holes is present in the sensor model, which helps control dispersion and provide light guidance.
- A thin layer of noble metals is deposited on the inner surface of selected cladding holes or externally on the fiber surface.
- The plasmonic layer interacts with the evanescent tail of the guided mode, facilitating resonance excitation.
- These sensor models are easier to fabricate compared to more complex PCF designs like slotted- or elliptical-core PCFs.
- The presence of metal or metal–graphene composite coatings improve performance for specific sensing applications.

### 4.6. Multi-Air-Hole Cladding PCF-SPR Sensor

**Figure 6:**
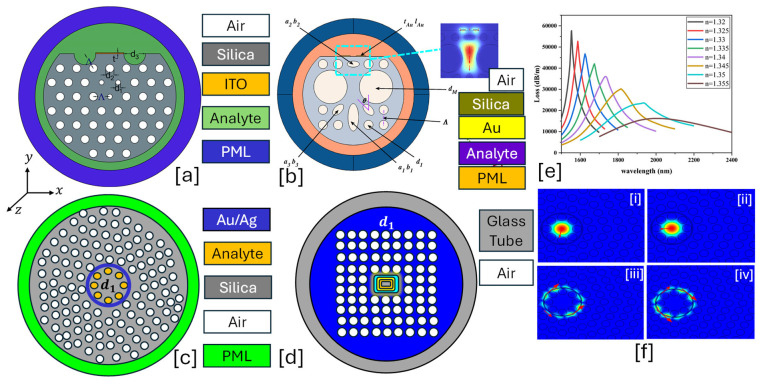
Multi-air holes cladding PCF-SPR sensor. (**a**) ITO-coated Quasi-D-shaped PCF having two slots in the background material [146]. (**b**) Au-coated PCF with circular and elliptical dimensions of air holes [147]. (**c**) Hexagonal lattice of air holes with analyte channel in the center of the PCF [148]. (**d**) Square lattice of the air holes with the core formation in the center of the PCF [149]. (**e**) CL behavior of the sensor model for RI ranging from 1.320 to 1.355 RIU for the sensor model presented in (**a**) [146]. (**f**) Mode profiles of the sensor model presented in (**c**) [148], [i] y-pol. core mode [148]; [ii] x-pol. core mode [148]; [iii] y-pol. SPP mode [148]; [iv] x-pol. SPP mode [148].

A multi-air-hole cladding PCF-SPR sensor is an advanced optical sensor that utilizes a cladding structure with multiple air holes to enhance light guidance and evanescent field interaction with the analyte. The presence of additional air holes in the cladding helps to engineer dispersion properties, improve mode confinement, and enhance sensing performance [144,145]. Figure 6 presents 2D models of multiple air holes in PCF-SPR sensors, which have a number of air holes in the background materials. The multi-air-hole cladding PCF-SPR sensor consists of the following features.

- A solid or hollow core, depending on the specific sensing requirement.
- A periodic array of multiple air holes arranged in a hexagonal, rectangular, or any geometrical lattice pattern, designed to control light propagation.
- A thin film of noble metals is deposited on inner air holes or externally coated on the fiber surface.

The multi-air-hole cladding PCF-SPR sensor exhibits superior performance due to enhanced light confinement and engineered dispersion properties. Some of the performance parameters of these sensors are listed as follows.

- Having a broad sensitivity range, depending on the air-hole configuration and plasmonic material.
- Can detect RI variations as low as 10^−6^ RIU, making it suitable for highly precise sensing.
- Moderate to low, as the optimized air-hole structure improves mode confinement while minimizing unnecessary losses.
- Typically designed to work in the VNIR range.

Some prominent features of these types of sensors are expressed as follows.

- The multi-air-hole structure enhances light confinement, leading to stronger evanescent field interactions.
- The presence of multiple air holes reduces propagation losses and improves sensor stability.
- Enhanced design reduces undesired coupling between different modes.

### 4.7. D-Shaped PCF-SPR Sensor

**Figure 7:**
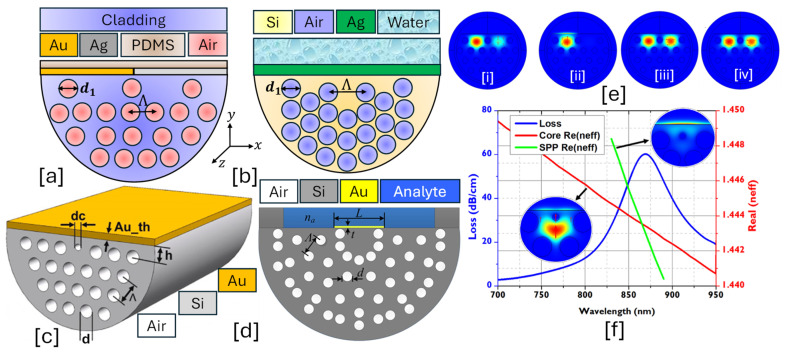
D-Shaped PCF-SPR sensor. (**a**) Dual metallic Au-Ag-coated PCF [152]. (**b**) Ag-coated water-based PCF [153]. (**c**) 3D model of the Au-coated PCF [154]. (**d**) Au-coated PCF with compact analyte channel [155]. (**e**) Mode profile of the sensor model presented in (**a**) [152], [i] y-polarized dual-core mode [152]; [ii] y-polarized single core mode [152]; [iii] x-polarized symmetric dual-core mode [152]; [iv] x-polarized anti-symmetric dual-core mode [152]. (**f**) Optical field distribution profile of the sensor model presented in (**c**) concerning core mode, SPP mode, and CL spectrum [154].

D-shaped PCF-SPR sensor is a specialized optical fiber sensor where a portion of the fiber cladding is polished into a flat surface, allowing for direct deposition of a plasmonic layer. This design enhances the interaction between the evanescent field and surface plasmons, leading to improved sensing performance [150,151]. The D-shaped structure provides a large surface area for analyte interaction, making it an excellent choice for applications. Figure 7 presents 2D and 3D models of D-shaped PCF-SPR sensors with a polished flat surface in the sensor model.

The D-shaped PCF-SPR sensor model consists of the following features.

- A section of the cladding is removed, creating a flat surface for easy metallic coating deposition to ensure proper mode confinement and dispersion control.
- A thin layer of noble metals or graphene–metal hybrids is deposited on the D-shaped surface to enable SPR excitation.
- The flat-polished D-shape enhances light–analyte interaction by bringing the core-guided mode closer to the plasmonic surface.

The D-shaped PCF-SPR sensor is known for its high sensitivity and efficient light–analyte interaction due to the reduced fiber thickness in the sensing region.

- The WS of these sensors can reach up to 50,000 nm/RIU or even higher, depending on the plasmonic material and fiber design.
- These sensors can detect RI changes as low as 10^−6^ RIU, making them suitable for ultra-precise sensing applications.
- D-shaped sensor models, due to their precise polishing, provide a strong plasmonic coupling.
- These sensors typically work within the VNIR range but are tunable to adjust the metal layer thickness and RI.

### 4.8. Internal Metal Deposition (IMD)-Shaped PCF-SPR Sensor

**Figure 8:**
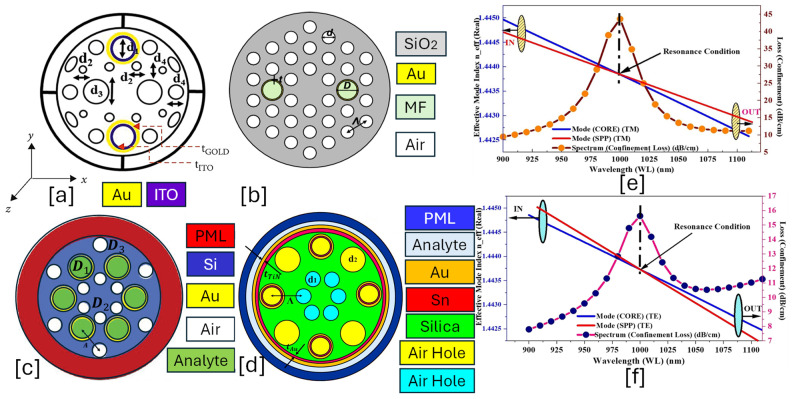
IMD-shaped PCF-SPR sensor. (**a**) Au-ITO-coated PCF with a combination of circular and elliptical air holes [159]. (**b**) Hexagonal lattice of Au-MF-coated PCF [160]. (**c**) Hexagonal lattice of Au-coated PCF with two dimensions of air holes [161]. (**d**) Au-Sn-coated PCF with two dimensions of air holes [162]. (**e**) TM-polarized optical field dispersion profile between core and SPP mode for the sensor model presented in (**d**) [162]. (**f**) TE-polarized optical field dispersion profile for the sensor model presented in (**d**) [162].

IMD-shaped PCF-SPR sensor is an advanced optical fiber sensor where the plasmonic material is coated on the inner walls of the air holes rather than on the fiber’s external surface [156]. This design enhances light–metal interaction, optimizes mode confinement, and reduces propagation loss, leading to superior sensing performance. IMD-based PCF-SPR sensors are particularly useful for biochemical, gas, and environmental sensing applications due to their high sensitivity, reduced loss, and improved resonance characteristics [157,158]. Figure 8 presents 2D models of IMD-shaped PCF-SPR sensors with an internal coating of plasmonic material on the air holes of the sensor model.

The IMD-shaped PCF-SPR sensor consists of the following features.

- These sensors can be solid or hollow, depending on the sensing application.
- Includes air holes, where a thin metallic layer is internally deposited to facilitate SPR excitation.
- Metal films are precisely coated inside the air-hole walls rather than on the fiber’s outer surface, enhancing plasmonic interaction with guided light.
- The IMD technique ensures stronger overlap between the guided mode and SPWs, leading to enhanced sensitivity and sharper resonance peak dips.

The advantages of the IMD-shaped PCF-SPR sensor are as follows.

- These models enhanced light–analyte interaction compared to external metal coatings.
- Unlike D-shaped sensors, the IMD approach keeps the fiber intact and assists in reducing signal attenuation.

### 4.9. External Metal Deposition (EMD)-Based PCF-SPR Sensor

**Figure 9:**
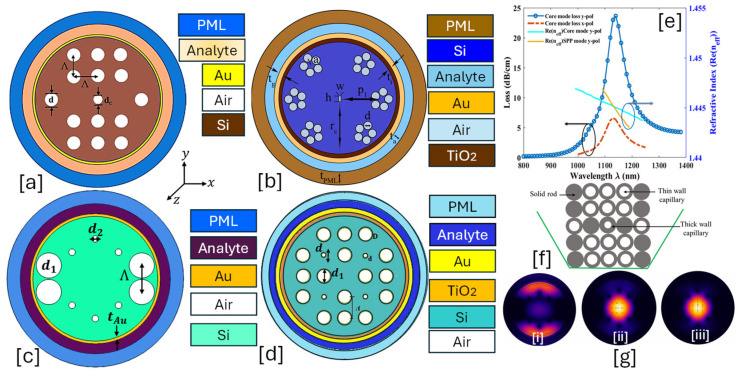
EMD-based PCF-SPR sensor. (**a**) Au-coated PCF with 2D air holes [165]. (**b**) Au-TiO_2_-coated PCF with a hexagonal lattice of air holes and a single lattice containing a combination of five air holes in a flower-shaped geometry [166]. (**c**) Au-coated PCF with two-dimensional air holes [167]. (**d**) Au-coated PCF with a modified octagonal lattice of air holes [168]. (**e**) Phase-matching condition for the sensor model presented in the (**a**) corresponding to both x-pol. and y-pol. [165]. (**f**) Stack-preform representation of the sensor model presented in (**b**) [166]. (**g**) Mode profile of the sensor model presented in (**c**) [167], [i] SPP mode [167]; [ii] x-polarized core mode [167]; [iii] y-polarized core mode [167].

A PCF-SPR sensor based on the EMD approach is a design where a metallic layer is applied to the external surface of the fiber, coating either the entire cladding or specific regions, creating a plasmonic layer for resonance excitation [163,164]. The EMD allows for improved light–analyte interaction, making it an effective solution for high-sensitivity biosensing, chemical detection, and environmental monitoring. Figure 9 presents 2D models of EMD-shaped PCF-SPR sensors with a coating of plasmonic material on the external body of the sensor surface. The EMD-based PCF-SPR sensor generally includes the following components.

- Typically, a solid or hollow core fiber, with the core size influencing the mode confinement and sensitivity.
- The cladding region consists of a microstructured air-hole lattice to control the light propagation, ensuring efficient confinement of the optical mode.
- A thin plasmonic layer is deposited on the outer surface of the PCF cladding, either over the entire cladding or only along specific sections.
- The evanescent field of the guided mode couples with the surface plasmons on the external metal layer, leading to SPR excitation at the interface between the metal and the surrounding medium.

The EMD-based PCF-SPR sensor offers excellent performance for various sensing applications. Key performance parameters of these sensor models are listed as follows.

- The sensitivity can range from 15,000 nm/RIU to 60,000 nm/RIU or even more, depending on the type of plasmonic metal and sensor structure.
- The sensor can detect small changes in RI with a resolution in the order of 10^−6^ RIU, making it suitable for ultra-sensitive applications.
- The CL is primarily determined by the metal deposition quality and fiber design. The EMD process may lead to higher propagation losses than IMD, but these losses can be compensated by careful optimization of the metal layer thickness.
- Typically designed to work in the visible to NIR range, tunable by adjusting plasmonic material thickness and fiber structure.

*Advantages*:

- The EMD sensor model is simpler to fabricate compared to other methods like IMD or D-shaped fibers.
- The evanescent field interaction with the plasmonic layer at the fiber’s surface is highly efficient for SPR sensing.
- The metal coating can be applied to specific regions of the fiber, allowing for customizable sensor designs.
- EMD methods are generally more cost-effective compared to alternative metal deposition techniques due to fewer complications during fabrication.

## 5. Current and Future Prospects of the PCF-SPR Sensor

### 5.1. Current Innovation in PCF-SPR Sensor

PCF-SPR biosensors have emerged as powerful tools for highly sensitive and label-free detection of biological and chemical substances. The synergy between PCF’s unique light-guiding capabilities and SPR’s resonance-based sensing mechanism allows for superior sensitivity, making them ideal for applications in biomedical diagnostics, environmental monitoring, and industrial sensing. Various configurations, such as core-excited, cladding-excited, D-shaped, side-polished, hollow-core, multi-core, IMD-shaped, EMD-shaped, and hybrid structures, have been developed, each offering specific advantages in terms of light confinement, interaction length, and ease of fabrication. The selection of plasmonic materials plays a crucial role in enhancing sensing performance, with conventional metals such as Au, Ag, Al, and Cu being widely used due to their strong plasmonic properties, while bimetallic and multilayer structures, dielectric coatings, and advanced and noble 2D materials like graphene, MXenes, TCOs, TMDs, and black phosphorus are being explored for improved stability and sensitivity. These sensors are extensively used in biomedical applications for detecting cancer biomarkers, viruses, glucose levels, RNA, and DNA hybridization, while in environmental monitoring, they facilitate the detection of heavy metal ions, pesticide residues, toxic gases, and pollutants. Additionally, they find applications in food safety analysis, pharmaceutical research, and industrial security for hazardous substance detection.

### 5.2. Application of PCF-SPR Sensor for Biomedical Applications

PCF-SPR biosensors have shown immense potential in a wide range of real-world applications due to their high sensitivity, tunability, and ability to detect minute changes in the RI [169]. These sensors are increasingly used for the detection of biomolecules such as DNA, proteins, glucose, and cancer biomarkers. For instance, PCF-SPR sensors functionalized with complementary DNA probes can detect specific nucleotide sequences or gene mutations, which is crucial for genetic screening and early disease diagnosis. Similarly, these sensors have been employed for protein and antigen detection, including key disease markers like C-reactive protein (CRP), prostate-specific antigen (PSA), and troponin, enhancing the early diagnosis of cardiovascular diseases and cancer [170]. In cancer diagnostics, PCF-SPR platforms integrated with advanced plasmonic materials like graphene oxide have been used to detect circulating tumor DNA and other tumor-specific markers with high accuracy. These sensors are also effective in monitoring blood glucose and cholesterol levels by immobilizing enzymes such as glucose oxidase or cholesterol oxidase onto the plasmonic surface, enabling real-time metabolic monitoring. Moreover, PCF-SPR biosensors are being developed for rapid virus detection, including pathogens such as SARS-CoV-2 and HIV, by incorporating surface-bound antibodies to identify viral proteins in clinical samples [171,172]. Beyond biomedical applications, these sensors are also useful in environmental monitoring for detecting heavy metal ions, pesticides, and microbial contaminants in water. By tailoring the PCF geometry and choosing appropriate plasmonic materials, these sensors can be optimized for specific analytes, making them highly adaptable tools for healthcare, biotechnology, food safety, and environmental analysis.

### 5.3. Challenges in PCF-SPR Sensor Fabrication

Despite their vast potential, challenges such as fabrication complexities, long-term stability, cost-effectiveness, and field deployment remain critical hurdles that must be addressed for widespread commercial adoption. With continuous advancements in optical fiber technology, photonics, and bioengineering, PCF-SPR biosensors are set to revolutionize optical sensing, offering unprecedented accuracy and efficiency across multiple disciplines, paving the way for next-generation biosensing platforms with enhanced reliability and real-time monitoring capabilities.

Fabrication difficulties

- Complex PCF structures
  - PCF-based sensors often require intricate designs such as D-shaped fibers, slotted-cores, or air-hole arrays with submicron precision [173].
  - Fabricating these structures with consistent geometry and minimal deviation is highly challenging and expensive, especially when alignment and symmetry are critical to sensor performance [173].
- Precision in metal deposition
  - SPR sensing requires accurate deposition of plasmonic materials, e.g., Au, Ag, etc., on the PCF structure, either internally—i.e., within air holes—or externally—i.e., on polished surfaces [174].
  - Techniques such as electron beam evaporation, sputtering, or chemical vapor deposition require high vacuum, cleanroom conditions, and specialized substrates, limiting scalability and increasing cost [174].
- Fragility and alignment issues
  - PCFs are mechanically delicate, and the post-processing steps like polishing, cleaving, or tapering for sensor preparation can easily cause structural damage [175].
  - Maintaining precise alignment between the PCF and external elements—e.g., analyte channels or microfluidics—is difficult and requires micron-level accuracy [175].

### 5.4. Addressing Fabrication Challenges in PCF-SPR Sensor Development

The future of PCF-SPR biosensors lies in the integration of advanced fabrication techniques such as 3D printing, nanofabrication, and laser-assisted etching to enhance precision and reproducibility, alongside the development of flexible and wearable biosensing platforms.

- Femtosecond laser micromachining
  - Allows high-precision structuring of PCFs, including D-shaped and slotted-geometries, with minimal thermal damage [176].
  - Enables direct writing of complex patterns into glass substrates with submicron accuracy, enhancing reproducibility and scalability [176].
- 3D nano printing and two-photon polymerization (2PP)
  - Advanced additive manufacturing techniques that can fabricate microstructured templates or fiber holders for better alignment [177].
  - Useful for developing customized sensor housings and on-fiber plasmonic elements [177].
- Atomic layer deposition (ALD)
  - Enables uniform, conformal coating of thin metal films inside air holes or on curved fiber surfaces with angstrom-level control, essential for SPR excitation [178].

### 5.5. Integration with Lab-on-Chip (LOC) and Microfluidic Systems

The integration of advanced plasmonic materials, novel geometries, and hybrid sensing approaches continues to improve their performance. Future developments in nanotechnology, AI-driven sensing, and flexible materials will further expand their applications, making them indispensable for next-generation optical biosensing solutions.

- Embedding PCF-SPR sensors into lab-on-chip platforms enables automated fluid handling, controlled analyte delivery, and high-throughput testing [179].
- Such integration will make biosensors compatible with point-of-care (POC) devices, allowing decentralized diagnostics in clinical and field settings [179].
- LOC integration also enhances sample specificity and sensor reusability by supporting multiplexing and functionalization in a confined, controlled environment.

### 5.6. AI-Assisted Biosensing and Smart Diagnostics

Artificial intelligence and machine learning are expected to play a transformative role in real-time data processing, pattern recognition, and predictive diagnostics, while the exploration of novel plasmonic materials such as topological insulators, quantum dots, and metamaterials is expected to further broaden their operational wavelength range and detection accuracy.

- Machine learning (ML) and deep learning (DL) are poised to transform biosensing through real-time data processing, pattern recognition, and analyte classification.
- AI models can analyze large volumes of spectral or resonance data to identify trends that may not be visible through conventional signal analysis [180].
- Advanced algorithms such as convolutional neural networks (CNNs) or support vector machines (SVMs) can predict disease signatures, quantify biomarker concentrations, and correct for noise and drift, making diagnostics more accurate and automated [180].

Finally, PCF-SPR biosensors have emerged as a promising technology for high-sensitivity biosensing across multiple fields. The integration of advanced plasmonic materials, novel geometries, and hybrid sensing approaches continues to improve their performance. Future developments in nanotechnology, AI-driven sensing, and flexible materials will further expand their applications, making them indispensable for next-generation optical biosensing solutions.

Table 1 presents a summarized overview of various key features of the PCF-SPR sensor and their applications in the optical world.

The standardization of PCF-based SPR sensors is an ongoing process, since it is still in its early stages compared to more mature sensing technologies. As research in this field rapidly advances, efforts are being made to develop consistent protocols for evaluating sensor performance parameters such as sensitivity, resolution, detection limit, and FOM. However, due to the wide variety of PCF geometries, plasmonic material combinations, and operating conditions like wavelength ranges and polarization modes, creating a universal standard remains a complex challenge. Currently, most studies use custom-designed models and report results under laboratory-specific conditions, which makes cross-comparison difficult. To address this, researchers are increasingly emphasizing reproducibility, modeling verification, e.g., via FEM or FDTD simulations, and alignment with real-world applications like biosensing or environmental monitoring. Scientific organizations have not yet released dedicated standards specifically for PCF-SPR sensors, but discussions within photonics and biosensing communities point toward a growing interest in defining performance benchmarks. As these sensors progress toward commercialization, standardization will become essential for quality control, regulatory approval, and interoperability, ultimately accelerating their integration into practical devices and diagnostic platforms [229,230].

## 6. Conclusions

PCF-SPR sensors represent a powerful and versatile platform that merges the structural tunability of PCFs with the high sensitivity of SPR. Through different core and cladding designs, along with strategic plasmonic material integration, these sensors offer superior control over light confinement and propagation, making them highly effective for detecting a wide range of biological and chemical analytes. Their demonstrated capability in sensing targets such as glucose, cholesterol, DNA, proteins, and cancer biomarkers highlights their potential in biomedical diagnostics and environmental monitoring. With ongoing advancements in nanofabrication techniques, plasmonic material engineering, and computational modeling, PCF-SPR sensors can be further optimized for higher sensitivity, broader RI detection, and multiplexed sensing. Their compatibility with miniaturized, lab-on-chip systems positions them as promising candidates for real-time, point-of-care diagnostics. As the demand for precise, label-free, and rapid biosensing continues to grow, PCF-SPR sensors play a transformative role across clinical, industrial, and research domains. Thus, PCF-SPR sensors are expected to play a critical role in advancing diagnostic and analytical techniques across multiple scientific and industrial domains.

## Figures and Tables

**Table 1:** Summarized overview of the PCF-SPR sensors.

S. No	Category	Details
1	Technology	PCF-SPR-based biosensors [181,182]
2	Advantage	High sensitivity [183], label-free detection [184], strong LMI [185]
3	PCF configurations	Core-excited [186], cladding-excited [187], D-shaped [188], side-polished [189], hollow-core [190], multi-core [191], hybrid structures [192]
4	Plasmonic materials	Au, Ag, Al, Cu [193], noble metals [194], TCOs [195], TMDs [196], bimetallic/multilayer coatings [197], graphene [198], MXenes [199], black phosphorus [200], etc.
5	Biomedical applications	Cancer biomarker detection [201], viruses [202], bacteria [203], glucose levels [204], urine sample analysis [205], µRNA [206], DNA hybridization [207]
6	Environmental applications	Heavy metal ion detection [208], pesticide residues [209], toxic gases detection [210], pollutants monitoring [211]
7	Other applications	Food safety [212], pharmaceutical research [213], industrial security [214], forensic investigation [215], hazardous substance detection [216]
8	Future trends	3D printing [217], nanofabrication [218], laser-assisted etching [219], AI and machine learning integration [220]
9	Emerging materials	Topological insulators [221], quantum dots [222], metamaterials [223]
10	Challenges	Fabrication complexities [224], long-term stability [225], cost-effectiveness [226], field deployment [227]
11	Potential impact	Next generation biosensing platforms with real-time monitoring and high reliability [228]

## Data Availability

The raw data supporting the Section 6 of this article will be made available by the authors on request.

## Abbreviations

The following abbreviations are used in this manuscript:

PCF	Photonic crystal fiber
SPR	Surface plasmon resonance
RI	Refractive index
IMD	Internal metal deposition
EMD	External metal deposition
PBG	Photonic bandgap
1D	One dimensional
2D	Two dimensional
3D	Three dimensional
Au	Gold
Ag	Silver
Al	Aluminum
Cu	Copper
Pt	Platinum
Pd	Palladium
UV	Ultraviolet
DNA	Deoxyribonucleic acid
VCO	Volatile organic compounds
CL	Confinement loss
B	Birefringence
WS	Wavelength sensitivity
AS	Amplitude sensitivity
SR	Sensor resolution
FOM	Figure of merit
S	Sensitivity
DA	Detection sensitivity
SPP	Surface plasmon polariton
VNIR	Transparent conducting oxides
ITO	Indium tin oxide
AZO	Aluminum-doped zinc oxide
GZO	Gallium-doped zinc oxide
FTO	Fluorine-doped tin oxide
CdO	Cadmium oxide
MIR	Mid infrared
LSPR	Localized surface plasmon resonances
TMD	Transition metal dichalcogenides
MoS2	Molybdenum disulfide
WS2	Tungsten disulfide
MoSe_2_	Molybdenum diselenide
WSe2	Tungsten diselenide
M-TIR	Modified total internal reflection
ARG	Anti-resonant guiding
